# Prevalence, causes, impacts, and management of needle phobia: An international survey of a general adult population

**DOI:** 10.1371/journal.pone.0276814

**Published:** 2022-11-21

**Authors:** Kimberly Alsbrooks, Klaus Hoerauf

**Affiliations:** 1 Becton, Dickinson, and Company, Franklin Lakes, NJ, United States of America; 2 Medical University of Vienna, Vienna, Austria; University of Catania Libraries and Documentation Centre: Universita degli Studi di Catania, ITALY

## Abstract

Needle phobia is an overlooked condition that affects virtually all medical procedures. Our study aimed to identify how commonly needle phobia is experienced, its underlying reasons, impacts, and potential mitigation strategies. A global survey was conducted in a general adult population using a questionnaire based on a targeted literature review that identified under-researched areas. The 21-item questionnaire was completed on a secure, web-based survey platform. Statistical analyses and models were utilized to identify relationships between participant characteristics and needle phobia. Of the 2,098 participants enrolled in the study, 63.2% (n = 1,325) reported experiencing needle phobia, and rated the intensity of their fear as 5.7 (±2.6) on average on a scale from 0 (no fear) to 10 (very strong/unreasonable fear or avoidance). According to the logistic regression model, other medical fears (odds coefficient = 2.14) and family history (1.67) were the most important factors associated with needle phobia. General anxiety (96.1%) and pain (95.5%) were the most common reasons for needle fear. Of the participants experiencing needle phobia, 52.2% stated avoiding blood draws, followed by 49.0% for blood donations, and 33.1% for vaccinations. While 24.3% of participants have seen a therapist, most have never sought help. The majority have shared their fear with nurses (61.1%) or physicians (44.4%); however, the provider helpfulness was rated as 4.9 (±3.1) on average on a scale from 0 (unhelpful) to 10 (extremely helpful). Utilizing non-invasive alternatives (94.1%) and smaller needles (91.1%) were most commonly identified as potential device-related solutions to alleviate fear; distractions (92.1%) and relaxation techniques (91.7%) were the top non-device-related approaches. Our findings highlight the prevalent nature of needle phobia and provide insights into its etiology and effects on patient care. Clinician responses were not perceived as helpful, emphasizing the need to address needle phobia, and improve patient experience.

## Introduction

Needles are routinely used in various clinical settings [1] to enable the delivery of drugs, vaccines, and other substances into the body or for fluid and tissue extraction, [2] and billions of injections are administered annually worldwide according to the WHO [3]. Needle phobia is an understudied condition, [4] which is experienced by patients undergoing procedures such as venipunctures and blood donations as well as those with chronic conditions necessitating frequent injections [5–8]. Needle fear exists on a continuum of severity, and can lead to delayed therapy, treatment avoidance, and vaccine hesitancy [6, 7, 9–13].

A significant portion of literature regarding the prevalence and severity of needle-phobia focused on specific subpopulations (i.e., pregnant patients, children, travelers), [5, 14, 15] and the limited research in the general population reports a wide range of prevalence rates from 2.1% to 30%. While a US-based survey reported a remarkably low prevalence (2.1%) of blood-injection-injury phobia (BII; i.e., fear of seeing blood or getting an injection) in adults, [16] a more recent review of the literature showed a needle fear prevalence rate of 20‐30% in the 20–40 age group [8].

Multiple innovation-related (e.g., using smaller/thinner needles or autoinjectors) [17–21] and other strategies (e.g., educational and psychological interventions) [22, 23] to address needle phobia were presented in the literature. However, these assessments were generally limited to evaluating a single approach without comparing different approaches/strategies to address needle fear. Furthermore, patient preferences were not considered, which may provide essential input to developing tailored approaches that address patients’ needs [24]. It is crucial to engage with patients, and integrate their perspectives into the clinical practice and product life cycle of medical devices [24]. In fact, the FDA recognized the importance of patient perspectives and began to incorporate patient input into the medical device evaluation processes about a decade ago [24].

There is a need to investigate the prevalence and severity of needle phobia in a general adult population, and capture patient perspectives regarding strategies that can be utilized to address needle fear. This study aims to identify how commonly and to what extent needle phobia is experienced by a global adult population, its underlying reasons, impacts, and potential strategies to alleviate it.

## Methods

### Study design and participants

A global survey-based study was conducted in a general adult population in January 2022. This study was determined to be exempt from local institutional review board (IRB) review in advance by the WCG IRB (Puyallup, WA).

Participants were recruited using the convenience sampling method, and any adults willing and able to complete the questionnaire were considered potential study subjects. A total of 2,000 subjects were anticipated to participate in the study. Informed consent was acquired through a written consent form, which was sent to potential participants along with the study details and the research team’s contact information. Subjects interested in participating in the study electronically completed the consent form to be able to proceed to the questionnaire. The consent information for all the participants was collected by the electronic survey platform that was utilized for the study.

### Questionnaire content & administration

A 21-item questionnaire was utilized and is provided in S1 Appendix. The questionnaire consisted of four main sections and included multiple-choice, 11-point Likert-like scale, ranking, and open-ended questions. The first section assessed how common needle phobia is, its underlying reasons, and its impacts on overall well-being. The second section covered mitigation strategies to identify potential approaches that may be used to alleviate the fear of needles. The third and fourth sections included background questions regarding demographics and overall perception of medical care.

The contents of the questionnaire were developed based on a comprehensive literature review, which identified under-researched areas, including variability in prevalence, underlying reasons, direct/downstream impacts, and approaches to alleviate needle fear. Database searches were limited to peer-reviewed manuscripts published between 2011–2021. Of a total of 334 studies that were identified, 263 papers were selected for full-text review after screening, and 163 were found to be relevant.

The questionnaire was hosted on a secure, web-based survey platform (i.e., SurveyMonkey). Responses to the questionnaire were encrypted and submitted anonymously.

### Analyses and statistical models

The minimum sample required to achieve a 95% confidence interval and a 2.5% error margin was 1,573. Expecting a 20% drop-out, 2,000 participants was the enrollment target for the study.

Various statistical techniques were utilized to evaluate the data collected by the surveys. The questions were categorized into three different groups: (i) participant characteristics, (ii) alleviation strategies, and (iii) intensity, potential causes, and impacts. Participant characteristics consisted of binary and categorical features, while the other two groups consisted of multiple-choice, ranking, and Likert-scale questions. Counts and percentages were calculated for questions in all three groups, and statistical significances were determined for participant characteristics using chi-square tests [25]. Descriptive statistics (mean, standard deviation, interquartile range) were utilized for Likert-scale questions [26] to understand data distributions better.

**Fig 1:**
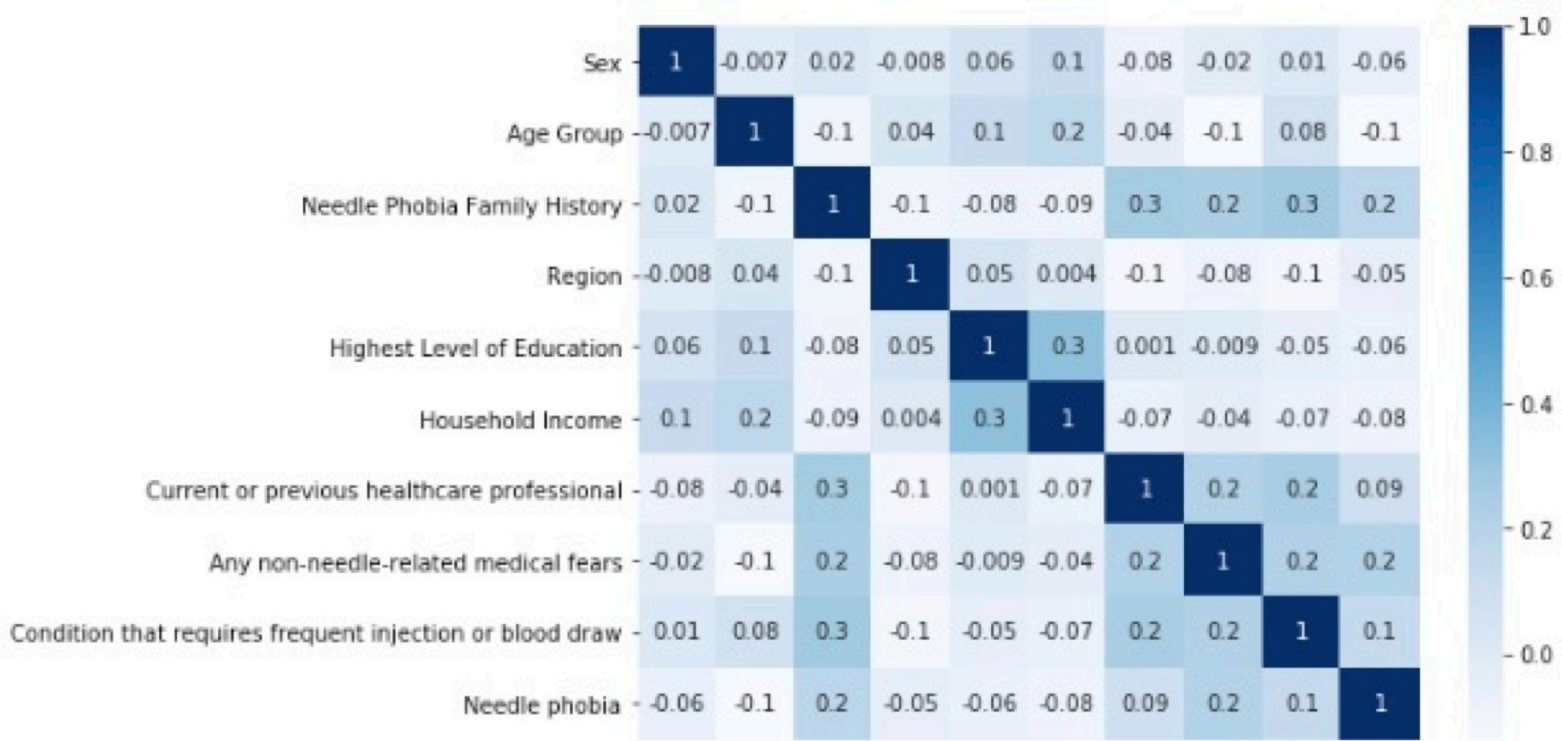
Correlation heat map.

Pre-processing was conducted as necessary to transform the dataset into the appropriate data formats for further analysis and modeling. Exploratory data analyses were performed to visualize general patterns present in the dataset. Data representing the participant characteristics were transformed from categorical to numeric values using binary and multi-class encoding (for binary and multi-class variables) and ordinal encoding (for ordinal variables) [27]. Descriptive statistics were acquired for all independent variables (i.e., participant characteristics), and the dependent variable (i.e., needle phobia), to understand their distributions. Correlations with respect to needle phobia were calculated for all variables. A heatmap with all variable correlations was used to visualize the relationships between the independent variables and the dependent variable (i.e., outcome), as well as the relationships among the independent variables (Fig 1). As a test for multicollinearity among the regressors, variance inflation factor (VIF) was calculated for all independent variables [28].

Statistical models were built using the transformed data and were optimized for accuracy in predicting needle phobia. The dataset was normalized into the range (0, 1) [29] and split into training and test sets [30]. Four algorithms were used to build initial models: Logistic Regression, K Nearest Neighbors, Decision Tree, and Random Forest [31]. Model evaluation was performed using a classification report for all initial models. Odds coefficients were calculated for the Logistic Regression model [32]. Feature importance was visualized for Logistic Regression, Decision Tree, and Random Forest models. Based on insights from exploratory data analyses and initial models, a final model was built, and Logistic Regression was selected as the algorithm for the final model. Numerous model combinations were tested, using grid search to exhaustively search for the best combination of parameter values and feature importance [33]. The final model was evaluated using odds coefficients for feature importance, 5-fold cross-validation, [34] confusion matrix, and classification report. The following equation was used for the final Logistic Regression model:

$$log\left(\frac{p}{1-p}\right)=a+bX_{1}+cX_{2}+dX_{3}+eX_{4}+fX_{5}+gX_{6}+hX_{7}+iX_{8}+jX_{9}$$

All statistical analyses were performed using Microsoft Excel and Python version 3.7.6. Python libraries used included: Pandas, Sklearn, Scipy, Matplotlib, and Seaborn.

## Results

### Prevalence of needle phobia and participant characteristics

A total of 2,098 participants completed the survey and 63.2% (n = 1,325) reported experiencing needle fear. Detailed participant characteristics are provided in Table 1. Most participants experiencing needle fear were female (n = 739; 55.8%; p = 0.004) and approximately half were in either 45–54 (n = 318; 24.0%) or 25–34 age groups (n = 292; 22.0%). The majority of participants with needle fear did not have family history (n = 897; 67.7%; p < 0.001) and had not worked as healthcare professionals (70.9%; n = 940; p < 0.001). Non-needle-related medical fears were experienced by 36.8% (n = 488) of participants with needle fear (p < 0.001) and 31.2% (n = 414; p < 0.001) had a condition that requires frequent injections or blood draws.

**Table 1:** Participant demographics and characteristics.

Characteristics	Total Participants (N = 2,098)		Participants with Fear of Needles				
			Yes (N = 1,325)		No (N = 773)		
	N	%	N	%	N	%	p-value*
**Sex**							0.004
Female	1120	53.4%	739	55.8%	381	49.3%	
Male	977	46.6%	585	44.2%	392	50.7%	
Missing	1	0.0%	1	0.1%	0	0.0%	
**Age group (years)**							<0.001
18 to 24	319	15.3%	230	17.4%	89	11.5%	
25 to 34	430	20.7%	292	22.0%	138	17.9%	
35 to 44	295	14.2%	190	14.3%	105	13.6%	
45 to 54	528	35.4%	318	24.0%	210	27.2%	
55 to 64	300	14.4%	183	13.8%	117	15.1%	
65 to 74	169	8.1%	85	6.4%	84	10.9%	
75 or older	39	1.9%	16	1.2%	23	3.0%	
Missing	18	0.9%	11	0.8%	7	0.9%	
**Geographic region**							0.214
North America	1,848	88.1%	1,153	87.0%	695	89.9%	
Middle East	60	2.9%	44	3.3%	16	2.1%	
Asia	47	2.2%	34	2.6%	13	1.7%	
Europe	41	2.0%	31	2.3%	10	1.3%	
South America	41	2.0%	27	2.0%	14	1.8%	
Oceania	15	0.7%	8	0.6%	7	0.9%	
Missing	46	2.2%	28	2.1%	18	2.3%	
**Highest level of education**							<0.001
4-Year College Degree	582	27.7%	360	27.4%	222	28.7%	
Some College, but no Degree	406	19.4%	247	18.8%	159	20.6%	
High School Diploma (or GED)	363	17.3%	248	18.9%	115	14.9%	
Graduate Level Degree	350	16.7%	195	14.9%	155	20.1%	
2-Year College Degree	246	11.7%	174	13.3%	72	9.3%	
Some High School, but no Diploma	81	3.9%	58	4.4%	23	3.0%	
Primary School	38	1.8%	28	2.1%	10	1.3%	
None of the Above	9	0.4%	2	0.2%	7	0.9%	
Missing	23	1.1%	13	1.0%	10	1.3%	
**Household income**							<0.001
$0-$9,999	200	9.5%	152	11.5%	48	6.2%	
$10,000-$24,999	243	11.6%	167	12.6%	76	9.8%	
$25,000-$49,999	442	21.1%	267	20.2%	175	22.6%	
$50,000-$74,999	365	17.4%	245	18.5%	120	15.5%	
$75,000-$99,999	275	13.1%	163	12.3%	112	14.5%	
$100,000-$124,999	153	7.3%	93	7.0%	60	7.8%	
$125,000-$149,999	81	3.9%	40	3.0%	41	5.3%	
$150,000-$174,999	43	2.0%	25	1.9%	18	2.3%	
$175,000-$199,999	14	0.7%	8	0.6%	6	0.8%	
$200,000+	91	4.3%	54	4.1%	37	4.8%	
Prefer not to answer	190	9.1%	110	8.3%	80	10.3%	
Missing	1	0.0%	1	0.1%	0	0.0%	
**Current or previous work as healthcare professional**							<0.001
Yes	459	21.9%	334	25.2%	125	16.2%	
No	1,545	73.6%	940	70.9%	605	78.3%	
Prefer not to answer	94	4.5%	51	3.8%	43	5.6%	
**Needle Phobia Family History**							<0.001
Yes	550	26.2%	428	32.3%	122	15.8%	
No	1,548	73.8%	897	67.7%	651	84.2%	
**Any non-needle-related medical fears**							<0.001
Yes	616	29.4%	488	36.8%	128	16.6%	
No	1341	63.9%	757	57.1%	584	75.5%	
Prefer not to answer	141	6.7%	80	6.0%	61	7.9%	
**Condition that requires frequent injection or blood draw**							<0.001
Yes	555	26.5%	414	31.2%	141	18.2%	
No	1445	68.9%	850	64.2%	595	77.0%	
Prefer not to answer	98	4.7%	61	4.6%	37	4.8%	

*Chi-squared test for categorical variables was used

Statistical methods, including regression models, were utilized to identify relationships between participant characteristics and needle phobia. Correlation values were low to moderate for the respective variables, ranging from approximately 0 to as high as 0.3 (Fig 1). The highest positively correlated independent variables with needle phobia were non-needle-related medical fears (correlation coefficient: 0.21), family history of needle phobia (0.18), and presence of a condition that requires frequent injection or blood draw (0.14). The highest negatively correlated independent variables with needle phobia were age group (-0.14), household income (-0.08), and the highest level of education (-0.06). VIF values for all independent variables were within an acceptable range (< 5), which indicated that multicollinearity was not an issue.

**Fig 2:**
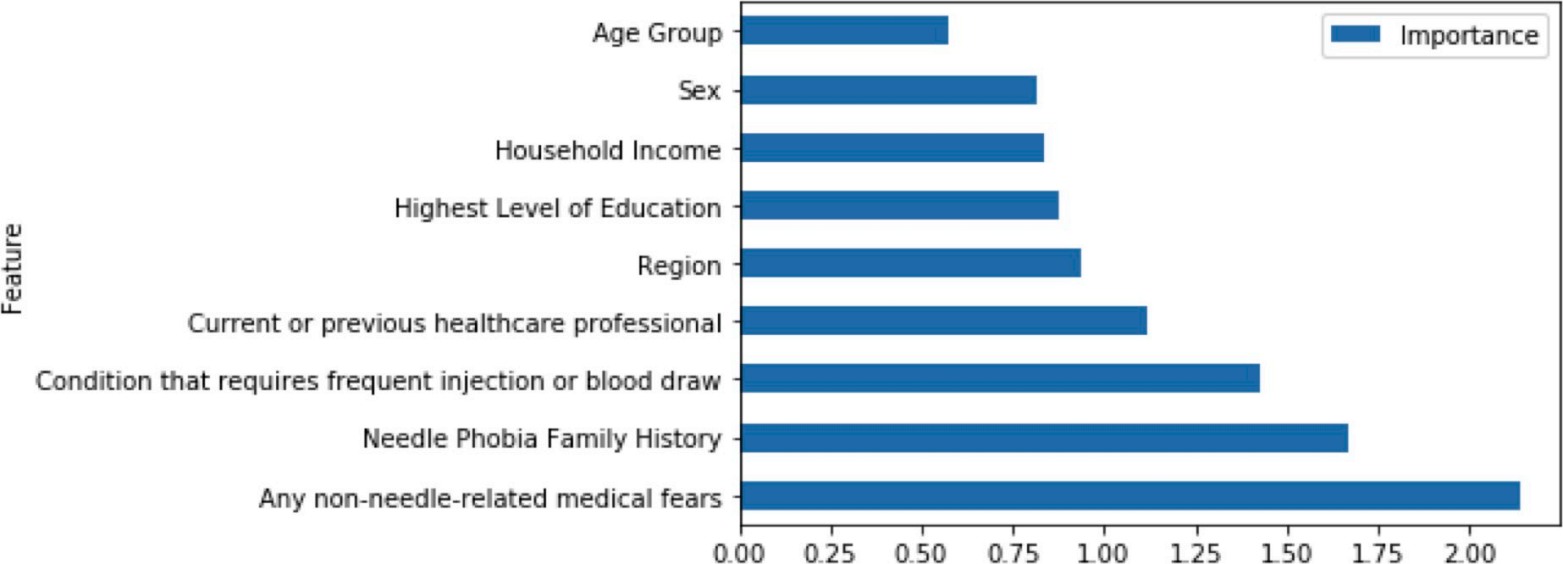
Feature importance (odds coefficients) bar chart for final logistic regression model.

Results for the final model were summarized by odds coefficients for feature importance (Fig 2), confusion matrix (S1 Fig), and classification report (S2 Fig). The most important features, according to the Logistic Regression model, were non-needle-related medical fears (odds coefficient = 2.14), family history of needle phobia (1.67), and presence of a condition that requires frequent injections or blood draws (1.43). The model had 64% overall accuracy on the training data, and 66% precision, 87% recall, and 75% f1 score for the positive class. For the testing data, the model had 67% accuracy overall, with 71% precision, 86% recall, and 78% f1 score for the positive class.

### Severity, underlying reasons, and impacts of needle phobia

A scale from 0 (no fear) to 10 (very strong/unreasonable fear or avoidance) was used to assess the intensity of needle fear experienced by the subjects. Participants with needle phobia provided an average rate of 5.7 (± 2.6) and a median [25th, 75th Percentiles] of 6.0 [4.0, 8.0] to describe the intensity of their fear before, during, and after a medical procedure/intervention.

Participant responses regarding the causes and impacts of needle phobia are reported in Table 2. General anxiety (n = 1,273; 96.1%) and pain (n = 1,266; 95.5%) before or during a medical procedure were the most common contributors to fear of needles. Pain was ranked by 37.1% of participants as the largest contributor to their needle fear.

**Table 2:** Causes and impacts of needle fear.

Causes and Impacts of Needle Fear	Participants with Needle Fear (N = 1,325)	
	N	%
**Contributors to needle fear experienced during or before medical procedures/interventions** *		
General anxiety	1,273	96.1%
Pain	1,266	95.5%
Fear of something going wrong during the procedure	1,262	95.2%
Fear of fainting/feeling dizzy	1,248	94.2%
Previous traumatic experience with needles	1,246	94.0%
Having to see blood	1,234	93.1%
Disgust regarding the procedure	1,232	93.0%
Other	1,082	81.7%
**Largest contributor to needle fear experienced during or before medical procedures/interventions**		
Pain	491	37.1%
General anxiety	304	22.9%
Previous traumatic experience with needles	232	17.5%
Having to see blood	66	5.0%
Fear of something going wrong during the procedure	65	4.9%
Fear of fainting/feeling dizzy	56	4.2%
Other	31	2.3%
Disgust regarding the procedure	29	2.2%
Procedures avoided to reduce exposure to needles*		
Blood draw from a vein in the arm	691	52.2%
Blood donation	649	49.0%
Vaccinations	439	33.1%
Injection for pain relief	416	31.4%
Injection for the treatment of a mild medical condition (low risk of morbidity)	359	27.1%
Capillary blood draw (fingerstick)	348	26.3%
Injection for the treatment of a severe medical condition (significant risk of morbidity/mortality)	242	18.3%

* Participants could select more than one option

Potential impacts of needle phobia on the participant behavior were also evaluated and provided in Table 2. Of the participants experiencing needle phobia, 52.2% (n = 691) stated avoiding blood draws, followed by 49.0% for blood donations (n = 649), 33.1% for vaccinations (n = 439). Injections to treat a severe medical condition were the least likely to be avoided by the participants (18.3%; n = 242).

### Strategies to alleviate needle phobia

Most participants (n = 988; 74.6%) had never sought help while 24.3% participants had seen a therapist either in person (n = 169; 12.8%) or remotely (n = 153; 11.5%). The majority of participants had shared their fear of needles with clinicians (nurses [n = 810; 61.1%]; physicians [n = 588; 44.4%]). The helpfulness of the providers was rated 4.9 (± 3.1) on average on a scale from 0 (unhelpful) to 10 (extremely helpful).

Participants experiencing needle phobia were also asked to indicate the strategies that would reduce their fear of needles and then rank their perceived effectiveness (Table 3). Non-invasive alternatives (n = 1,247; 94.1%) and smaller needles (n = 1,207; 91.1%) were most commonly identified as the device-related solutions to alleviate needle phobia. Smaller needles were ranked as the most helpful device to reduce needle fear by 32.6% of the participants, followed by non-invasive alternatives (26.4%) and autoinjectors (16.5%). Distractions (n = 1,220; 92.1%) and relaxation techniques (n = 1,215; 91.7%) were the most commonly selected non-device-related strategies, and distractions were identified as most helpful (35.9% of the participants experiencing needle phobia).

**Table 3:** Device and non-device-based strategies that would reduce needle fear of participants.

Strategies that would reduce needle fear	Participants with Needle Fear (N = 1,325)	
	N	%
**Devices** *		
Non-invasive alternatives	1,247	94.1%
Smaller needles	1,207	91.1%
Needle-free jet injectors	1,203	90.8%
Autoinjectors (i.e., invisible needles)	1,189	89.7%
Insulin delivery devices	1,180	89.1%
**Most helpful device**		
Smaller needles	432	32.6%
Non-invasive alternatives	350	26.4%
Autoinjectors (i.e., invisible needles)	219	16.5%
Needle-free jet injectors	202	15.2%
Insulin delivery devices	48	3.6%
**Non-device-based interventions** *		
Distractions during the procedure	1,220	92.1%
Relaxation techniques	1,215	91.7%
Using topical numbing creams	1,204	90.9%
Education/information on how the medical equipment works	1,174	88.6%
Consultations with the clinician regarding the importance of the procedure/treatment	1,155	87.2%
Seeing a therapist	1,147	86.6%
Watching blood draw videos before/during the procedure	1,139	86.0%
**Most helpful non-device-based intervention**		
Distractions during the procedure	476	35.9%
Education/information on how the medical equipment works	308	23.2%
Using topical numbing creams	183	13.8%
Relaxation techniques	138	10.4%
Seeing a therapist	74	5.6%
Watching blood draw videos before/during the procedure	38	2.9%
Consultations with the clinician regarding the importance of the procedure/treatment	25	1.9%

* Participants could select more than one option

## Discussion

Herein, we present a comprehensive survey-based evaluation of needle phobia in a large global adult population. We investigated the prevalence, underlying reasons, impacts, and potential management strategies for needle phobia. Our findings show that over two-thirds of the participants experience some level of needle fear, highlighting its widespread nature. This is significantly higher than the prevalence values reported in the literature for the general adult population (2.1% to 30%), [8, 16, 35, 36] which may partially be due to the variability in defining needle phobia.

Needle fear can be conceptualized on a continuum, from fear to more severe presentations and diagnoses of phobia, depending on the level of distress and impairment/interference [37]. In the Diagnostic and Statistical Manual of Mental Disorders (DSM), needle phobia was formally defined under the BII type of specific phobia [38]. However, the literature used various definitions/nomenclature interchangeably such as belonephobia (fear of needles and pins), trypanophobia (fear of injections), and aichmophobia (fear of sharp, pointed objects) [39]. In 2007, a large US-based survey evaluated the epidemiology of needle phobia using the DSM criteria and reported a strikingly low prevalence (2.1%) of BII phobia in the total adult population [16]. On the other hand, a review of the literature focusing on adults with different chronic diseases used a broader definition as discomfort, anxiety, fear, distress and/or phobia related to needles [22]. The study reported higher rates of needle fear, with prevalence ranging from 17% to 52% among adults with past or current experience of chemotherapy and from 25% to 47% among adults receiving peritoneal dialysis or hemodialysis [22].

While using a clear and universal definition of needle phobia can benefit clinical practice and research, any level of needle fear is important, considering its significant negative impacts on physical and mental well-being [6, 7, 9–13]. Adopting a broad definition may be more inclusive of patients experiencing needle fear in their daily lives, who may not fulfill the criteria for narrow definitions, and could still present risks of avoiding medical care [6, 7, 10–12]. Thus, the high prevalence reported in this study may be more representative of the burden needle phobia constitutes in the general adult population.

Our analyses revealed that non-needle-related medical fears and family history of needle phobia were the most important factors associated with needle phobia, which is consistent with the relevant literature. Individuals diagnosed with a specific phobia were previously shown to present with comorbid phobias [40]. Moreover, a familial tendency for needle phobia has been reported by Orenius et al., who stated that approximately 80% of patients with needle phobia report strong needle fear in a first-degree relative [39, 41].

Having a condition requiring frequent injections/blood draws was identified as another important factor in our regression model, which is particularly relevant considering distinct ways needle fear can alter overall well-being. First, patients with chronic or severe conditions may be more likely to develop needle phobia due to frequent and long-term exposure to needles as part of essential disease treatment. It was shown that the patients with frequent exposure to the medical system are more likely to have painful experiences and develop needle phobia [5, 17, 42]. For example, patients receiving hemodialysis typically require a minimum of 312 needle insertions per year, and needle fear was reported by 25–47% of adults receiving dialysis [22]. Second, needle phobia may lead to poor disease prognosis due to poor adherence to treatment. [10] Because these patients are likely suffering from chronic (e.g., kidney disease) or other severe conditions (e.g., cancer), they may be more vulnerable to needle phobia and its impacts on treatment adherence. Avoidance behavior may have a more significant impact on patients with conditions requiring frequent injections compared to healthier individuals [43]. In fact, literature reports adverse health outcomes and increased mortality in type II diabetes and hypertension patients with poor treatment adherence, which typically comprises frequent injections and blood draws [43].

Different potential reasons may be behind the emergence of needle phobia. While pain was identified as the largest contributor in our research, all the potential causes that we tested were selected by over 90% of participants experiencing needle phobia as contributors to their fear. This highlights the complex and multifactorial etiology behind needle phobia, which is consistent with the literature [10, 44]. Furthermore, it is not surprising that pain was the most critical reason considering a robust body of literature endorsing it as the most prominent underlying reason for needle phobia [5, 17, 42, 45].

As expected, needle fear was associated with widespread avoidance behavior. Most of the subjects with needle fear reported avoiding blood draws, and over one-third reported avoiding blood donations and vaccinations. Needle fear is a common barrier to initiating or adhering to medical treatments and vaccine hesitancy [9–13, 22]. An exploratory study in a tertiary care hospital reported that around half of diabetic patients delayed the start or avoided treatment due to the fear of needles and injections [10]. Moreover, in a school in Colombia, one-quarter of girls considered needle fear as a barrier to obtaining HPV vaccination [12]. Vaccine hesitancy is associated with significant public health implications: outbreaks of measles, mumps, rubella, and pertussis, were linked to under-vaccinated communities [46–48]. More recently, COVID-19 infection and death during the Delta-predominant period and Omicron emergence were higher among unvaccinated persons [49].

Although the treatment for severe conditions was less likely to be avoided (18.3% of participants with needle phobia), poor treatment adherence for these conditions may result in particularly consequential health outcomes [10]. Furthermore, patients experiencing needle phobia are likely more prone to become severely sick since avoiding treatment of a mild medical condition (low risk of morbidity) or vaccinations can lead to severe disease [10, 46].

While most participants have never sought outside help (i.e., therapy), they have shared their fear of needles with providers. The lack of perceived helpfulness of the providers (rated as 4.9 on a scale from 0 to 10) is concerning. Clinicians may benefit from education in identifying and addressing needle phobia [39].

Various strategies to reduce needle fear were presented in the literature and tested in this survey to understand patient preferences and perspectives regarding their comparative effectiveness. All strategies evaluated were considered effective by the participants (at least 86% of participants with needle phobia; Table 3), which is supported by studies that suggested the role of these approaches in alleviating needle fear [14, 50–52]. Smaller needles were identified as the most helpful device in reducing needle fear, followed by non-invasive alternatives. While there is no study directly evaluating the impact of these interventions on needle phobia, smaller needles and other device innovations were shown to minimize pain, which is closely linked with needle phobia. Three RCTs conducted in various population groups (i.e., adults who underwent arterial punctures, diabetic adults, pediatrics) showed that smaller/thinner needles could effectively decrease pain associated with injections [17–19]. The potential positive impacts of non-invasive alternatives on pain and patient satisfaction were also shown in RCTs [50, 53]. Moreover, distractions and medical equipment-related education were perceived as the most helpful non-device-related strategies by participants. While the literature is more limited relative to device-related strategies, previous studies suggested education and distraction-based techniques as potential components of needle phobia interventions. In a case study conducted in a chemotherapy outpatient unit, a combination of nursing interventions, including distractions, improved outcomes in a cancer patient with a reduction in the related anxiety and needle fear [51]. An observational study also demonstrated that multidisciplinary educational approaches during pregnancy significantly improved fear of self-injections [14]. Furthermore, a combination of psychoeducation and applied tension resulted in substantial BII fear reductions and a change in avoidance behavior in 70% of the patients undergoing blood draws [52].

Our study results highlight the extent of needle fear in a general adult population, different factors that contribute to needle fear associated with remarkable avoidance behavior, and the importance of adequate fear management considering potential consequences on the individuals’ well-being and public health. These findings also provide the basis for future studies to evaluate the clinical value of various mitigation strategies, including their impact on avoidance behavior and patient outcomes.

### Limitations

This study included several limitations. Most study participants were from North America, and therefore our results may not be generalizable to the overall global adult population. Selection bias could have arisen with an online survey, which requires computer literacy and internet access. This could explain the under-representation of the elderly in this survey, as access to the internet is generally lower in the older age group. As this study reports preferences and perceived values, the data collected is non-objective and opinion-based. Finally, the reasons for needle phobia that were tested in the questionnaire may not constitute the actual sources of patients’ fear and may be rationalizations of their phobia, which might explain the high frequency reported for each one of them (selected by over 90% of patients).

## Conclusions

Our findings illustrate a strikingly high prevalence of needle phobia in a sizeable global adult population. Avoidance behavior is common among patients with needle fear and can negatively impact the well-being of individuals and their communities. Patients did not perceive clinician responses as helpful, highlighting a need to devise, evaluate and implement strategies to alleviate needle phobia, and improve the patient experience.

## Supporting information

S1 Appendix Survey questionnaire. (DOCX)

S1 Table Model variable descriptions, coefficient names and values. (DOCX)

S1 Fig Confusion matrix for model on test data. (TIF)

S2 Fig Classification report for model on test data. (TIF)

S1 Dataset Survey dataset. (XLSX)

## References

[1] Safely Using Sharps (Needles and Syringes) at Home, at Work and on Travel: U.S. Food and Drug Administration; 2021 [updated 11/19/2021; cited 2022 May 8]. Available from: https://www.fda.gov/medical-devices/consumer-products/safely-using-sharps-needles-and-syringes-home-work-and-travel.

[2] Gill HS, Prausnitz MR. Does needle size matter? J Diabetes Sci Technol. 2007;1(5):725–9. doi: 10.1177/193229680700100517 ; PubMed Central PMCID: PMC2769648. 19885141 PMC2769648

[3] Hayashi T, Hutin YJ, Bulterys M, Altaf A, Allegranzi B. Injection practices in 2011–2015: a review using data from the demographic and health surveys (DHS). BMC Health Serv Res. 2019;19(1):600. Epub 20190827. doi: 10.1186/s12913-019-4366-9 ; PubMed Central PMCID: PMC6712605. 31455315 PMC6712605

[4] Abado E, Aue T, Okon-Singer H. Cognitive Biases in Blood-Injection-Injury Phobia: A Review. Front Psychiatry. 2021;12:678891. Epub 20210713. doi: 10.3389/fpsyt.2021.678891 ; PubMed Central PMCID: PMC8313757. 34326784 PMC8313757

[5] McMurtry CM, Pillai Riddell R, Taddio A, Racine N, Asmundson GJ, Noel M, et al. Far From "Just a Poke": Common Painful Needle Procedures and the Development of Needle Fear. Clin J Pain. 2015;31(10 Suppl):S3–11. doi: 10.1097/AJP.0000000000000272 ; PubMed Central PMCID: PMC4900413. 26352920 PMC4900413

[6] Dean BW, Hewitt SN, Begos MC, Gomez A, Messam LLM. An analysis of blood donation barriers experienced by North American and Caribbean university students in Grenada, West Indies. Transfus Apher Sci. 2018;57(1):40–5. Epub 20171122. doi: 10.1016/j.transci.2017.11.026 . 29249628

[7] Murtagh CM, Katulamu C. Motivations and deterrents toward blood donation in Kampala, Uganda. Soc Sci Med. 2021;272:113681. Epub 20210106. doi: 10.1016/j.socscimed.2021.113681 . 33524905

[8] McLenon J, Rogers MAM. The fear of needles: A systematic review and meta-analysis. J Adv Nurs. 2019;75(1):30–42. Epub 20180911. doi: 10.1111/jan.13818 . 30109720

[9] Love AS, Love RJ. Considering Needle Phobia among Adult Patients During Mass COVID-19 Vaccinations. J Prim Care Community Health. 2021;12:21501327211007393. doi: 10.1177/21501327211007393 ; PubMed Central PMCID: PMC8020217. 33813931 PMC8020217

[10] Sharma SK, Kant R, Kalra S, Bishnoi R. Prevalence of Primary Non-adherence with Insulin and Barriers to Insulin Initiation in Patients with Type 2 Diabetes Mellitus—An Exploratory Study in a Tertiary Care Teaching Public Hospital. Eur Endocrinol. 2020;16(2):143–7. Epub 20201006. doi: 10.17925/EE.2020.16.2.143 ; PubMed Central PMCID: PMC7572173. 33117446 PMC7572173

[11] Cherian V, Saini NK, Sharma AK, Philip J. Prevalence and predictors of vaccine hesitancy in an urbanized agglomeration of New Delhi, India. J Public Health (Oxf). 2022;44(1):70–6. doi: 10.1093/pubmed/fdab007 . 33594438

[12] Cordoba-Sanchez V, Tovar-Aguirre OL, Franco S, Arias Ortiz NE, Louie K, Sanchez GI, et al. Perception about barriers and facilitators of the school-based HPV vaccine program of Manizales, Colombia: A qualitative study in school-enrolled girls and their parents. Prev Med Rep. 2019;16:100977. Epub 20190822. doi: 10.1016/j.pmedr.2019.100977 ; PubMed Central PMCID: PMC6722392. 31508297 PMC6722392

[13] McLaughlin K, Manns B, Mortis G, Hons R, Taub K. Why patients with ESRD do not select self-care dialysis as a treatment option. Am J Kidney Dis. 2003;41(2):380–5. doi: 10.1053/ajkd.2003.50047 . 12552500

[14] Feitosa AC, Sampaio LN, Batista AG, Pinheiro CB. Frequency of fear of needles and impact of a multidisciplinary educational approach towards pregnant women with diabetes. Rev Bras Ginecol Obstet. 2013;35(3):111–6. doi: 10.1590/s0100-72032013000300004 . 23538469

[15] Noble LM, Farquharson L, O’Dwyer NA, Behrens RH. The impact of injection anxiety on education of travelers about common travel risks. J Travel Med. 2014;21(2):86–91. Epub 20131119. doi: 10.1111/jtm.12081 . 24251652

[16] Stinson FS, Dawson DA, Patricia Chou S, Smith S, Goldstein RB, June Ruan W, et al. The epidemiology of DSM-IV specific phobia in the USA: results from the National Epidemiologic Survey on Alcohol and Related Conditions. Psychol Med. 2007;37(7):1047–59. Epub 20070305. doi: 10.1017/S0033291707000086 . 17335637

[17] Kour G, Masih U, Singh C, Srivastava M, Yadav P, Kushwah J. Insulin Syringe: A Gimmick in Pediatric Dentistry. Int J Clin Pediatr Dent. 2017;10(4):319–23. Epub 20170227. doi: 10.5005/jp-journals-10005-1458 ; PubMed Central PMCID: PMC5789132. 29403222 PMC5789132

[18] Ibrahim I, Yau YW, Ong L, Chan YH, Kuan WS. Arterial puncture using insulin needle is less painful than with standard needle: a randomized crossover study. Acad Emerg Med. 2015;22(3):315–20. Epub 20150302. doi: 10.1111/acem.12601 . 25731215

[19] Valentini M, Scardapane M, Bondanini F, Bossi A, Colatrella A, Girelli A, et al. Efficacy, safety and acceptability of the new pen needle 33G × 4 mm. AGO 01 study. Curr Med Res Opin. 2015;31(3):487–92. Epub 20141210. doi: 10.1185/03007995.2014.993025 . 25469829

[20] Phillips JT, Fox E, Grainger W, Tuccillo D, Liu S, Deykin A. An open-label, multicenter study to evaluate the safe and effective use of the single-use autoinjector with an Avonex® prefilled syringe in multiple sclerosis subjects. BMC Neurol. 2011;11:126. Epub 20111014. doi: 10.1186/1471-2377-11-126 ; PubMed Central PMCID: PMC3213083. 21999176 PMC3213083

[21] Ziemssen T, Sylvester L, Rametta M, Ross AP. Patient Satisfaction with the New Interferon Beta-1b Autoinjector (BETACONNECT™). Neurol Ther. 2015;4(2):125–36. Epub 20151027. doi: 10.1007/s40120-015-0036-y ; PubMed Central PMCID: PMC4685867. 26662362 PMC4685867

[22] Duncanson E, Le Leu RK, Shanahan L, Macauley L, Bennett PN, Weichula R, et al. The prevalence and evidence-based management of needle fear in adults with chronic disease: A scoping review. PLoS One. 2021;16(6):e0253048. Epub 20210610. doi: 10.1371/journal.pone.0253048 ; PubMed Central PMCID: PMC8192004. 34111207 PMC8192004

[23] Mackereth P, Hackman E, Tomlinson L, Manifold J, Orrett L. ’Needle with ease’: rapid stress management techniques. Br J Nurs. 2012;21(14):S18–22. doi: 10.12968/bjon.2012.21.Sup14.S18 . 23252177

[24] Tarver ME, Neuland C. Integrating Patient Perspectives into Medical Device Regulatory Decision Making to Advance Innovation in Kidney Disease. Clin J Am Soc Nephrol. 2021;16(4):636–8. Epub 20210303. doi: 10.2215/CJN.11510720 ; PubMed Central PMCID: PMC8092069. 33658182 PMC8092069

[25] Connelly L. Chi-Square Test. MEDSURG Nursing. 2019;28(2):127–. PubMed PMID: 135960900. Language: English. Entry Date: 20190423. Revision Date: 20190425. Publication Type: Article. Journal Subset: Core Nursing.

[26] Boone HN, Boone DA. Analyzing Likert Data. The Journal of Extension. 2012;50.

[27] Potdar K, Pardawala TS, Pai CD. A Comparative Study of Categorical Variable Encoding Techniques for Neural Network Classifiers. International Journal of Computer Applications. 2017;175:7–9.

[28] Senaviratna N, Cooray T. Diagnosing Multicollinearity of Logistic Regression Model. Asian Journal of Probability and Statistics. 2019:1–9. doi: 10.9734/ajpas/2019/v5i230132

[29] Patro SGK, Sahu KK. Normalization: A Preprocessing Stage. ArXiv. 2015;abs/1503.06462.

[30] Tan J, Yang J, Wu S, Chen G, Zhao J. A critical look at the current train/test split in machine learning. ArXiv. 2021;abs/2106.04525.

[31] Kumari R, Srivastava S. Machine Learning: A Review on Binary Classification. International Journal of Computer Applications. 2017;160:11–5. doi: 10.5120/ijca2017913083

[32] Huang FL, Moon TR. What Are the Odds of That? A Primer on Understanding Logistic Regression. Gifted Child Quarterly. 2013;57(3):197–204. doi: 10.1177/0016986213490022

[33] Liashchynskyi P, Liashchynskyi P. Grid Search, Random Search, Genetic Algorithm: A Big Comparison for NAS2019.

[34] Wong T-T, Yeh P. Reliable Accuracy Estimates from k-Fold Cross Validation. IEEE Transactions on Knowledge and Data Engineering. 2020;32:1586–94.

[35] Hamilton JG. Needle phobia: a neglected diagnosis. J Fam Pract. 1995;41(2):169–75. . 7636457

[36] Bienvenu OJ, Eaton WW. The epidemiology of blood-injection-injury phobia. Psychol Med. 1998;28(5):1129–36. doi: 10.1017/s0033291798007144 . 9794020

[37] McMurtry CM, Taddio A, Noel M, Antony MM, Chambers CT, Asmundson GJ, et al. Exposure-based Interventions for the management of individuals with high levels of needle fear across the lifespan: a clinical practice guideline and call for further research. Cogn Behav Ther. 2016;45(3):217–35. Epub 20160323. doi: 10.1080/16506073.2016.1157204 ; PubMed Central PMCID: PMC4867871. 27007463 PMC4867871

[38] Diagnostic and statistical manual of mental disorders: DSM-5. American Psychiatric A, American Psychiatric Association DSMTF, editors. Arlington, VA: American Psychiatric Association; 2013.

[39] Orenius T, LicPsych, Säilä H, Mikola K, Ristolainen L. Fear of Injections and Needle Phobia Among Children and Adolescents: An Overview of Psychological, Behavioral, and Contextual Factors. SAGE Open Nurs. 2018;4:2377960818759442. Epub 20180314. doi: 10.1177/2377960818759442 ; PubMed Central PMCID: PMC7774419. 33415191 PMC7774419

[40] Ollendick TH, Ost LG, Reuterskiöld L, Costa N, Cederlund R, Sirbu C, et al. One-session treatment of specific phobias in youth: a randomized clinical trial in the United States and Sweden. J Consult Clin Psychol. 2009;77(3):504–16. doi: 10.1037/a0015158 . 19485591

[41] Sokolowski CJ, Giovannitti JA Jr., Boynes SG. Needle phobia: etiology, adverse consequences, and patient management. Dent Clin North Am. 2010;54(4):731–44. doi: 10.1016/j.cden.2010.06.012 . 20831935

[42] Cook LS. Needle Phobia. J Infus Nurs. 2016;39(5):273–9. doi: 10.1097/NAN.0000000000000184 . 27598066

[43] Losi S, Berra CCF, Fornengo R, Pitocco D, Biricolti G, Federici MO. The role of patient preferences in adherence to treatment in chronic disease: a narrative review. Drug target insights. 2021;15:13–20. doi: 10.33393/dti.2021.2342 34785884 PMC8591552

[44] Ngassa Piotie P, Wood P, Webb EM, Marcus TS, Rheeder P. Willingness of people with Type 2 diabetes to start insulin therapy: Evidence from the South African Tshwane Insulin Project (TIP). Diabetes Res Clin Pract. 2020;168:108366. Epub 20200811. doi: 10.1016/j.diabres.2020.108366 . 32791159

[45] Smith NB, Meuret AE. The role of painful events and pain perception in blood-injection-injury fears. J Behav Ther Exp Psychiatry. 2012;43(4):1045–8. Epub 20120421. doi: 10.1016/j.jbtep.2012.03.006 ; PubMed Central PMCID: PMC3577418. 22677208 PMC3577418

[46] Dubé E, Gagnon D, Ouakki M, Bettinger JA, Guay M, Halperin S, et al. Understanding Vaccine Hesitancy in Canada: Results of a Consultation Study by the Canadian Immunization Research Network. PLoS One. 2016;11(6):e0156118. Epub 20160603. doi: 10.1371/journal.pone.0156118 ; PubMed Central PMCID: PMC4892544. 27257809 PMC4892544

[47] Willis DE, Andersen JA, Bryant-Moore K, Selig JP, Long CR, Felix HC, et al. COVID-19 vaccine hesitancy: Race/ethnicity, trust, and fear. Clin Transl Sci. 2021;14(6):2200–7. Epub 2021/07/02. doi: 10.1111/cts.13077 . 34213073 PMC8444681

[48] Kofman A, Kantor R, Adashi EY. Potential COVID-19 Endgame Scenarios: Eradication, Elimination, Cohabitation, or Conflagration? Jama. 2021;326(4):303–4. doi: 10.1001/jama.2021.11042 . 34236382

[49] Johnson AG, Amin AB, Ali AR, Hoots B, Cadwell BL, Arora S, et al. COVID-19 Incidence and Death Rates Among Unvaccinated and Fully Vaccinated Adults with and Without Booster Doses During Periods of Delta and Omicron Variant Emergence—25 U.S. Jurisdictions, April 4-December 25, 2021. MMWR Morb Mortal Wkly Rep. 2022;71(4):132–8. Epub 20220128. doi: 10.15585/mmwr.mm7104e2 . 35085223 PMC9351531

[50] Fernando GJP, Hickling J, Jayashi Flores CM, Griffin P, Anderson CD, Skinner SR, et al. Safety, tolerability, acceptability and immunogenicity of an influenza vaccine delivered to human skin by a novel high-density microprojection array patch (Nanopatch™). Vaccine. 2018;36(26):3779–88. Epub 20180517. doi: 10.1016/j.vaccine.2018.05.053 . 29779922

[51] Mendonça AB, Pereira ER, Magnago C, Silva RMCRA, Martins AdO. Nursing process for a patient with needle phobia: a case study. Revista brasileira de enfermagem. 2020;73 4:e20190095. doi: 10.1590/0034-7167-2019-0095 32578738

[52] Wannemueller A, Fasbender A, Kampmann Z, Weiser K, Schaumburg S, Velten J, et al. Large-Group One-Session Treatment: A Feasibility Study of Exposure Combined With Applied Tension or Diaphragmatic Breathing in Highly Blood-Injury-Injection Fearful Individuals. Front Psychol. 2018;9:1534. Epub 20180821. doi: 10.3389/fpsyg.2018.01534 ; PubMed Central PMCID: PMC6110887. 30186206 PMC6110887

[53] Yılmaz N, Erdal A, Demir O. A comparison of dry needling and kinesiotaping therapies in myofascial pain syndrome: A randomized clinical study. Turk J Phys Med Rehabil. 2020;66(3):351–9. Epub 20200818. doi: 10.5606/tftrd.2020.3917 ; PubMed Central PMCID: PMC7557629. 33089092 PMC7557629

