# Supplementary material for: Prevalence, causes, impacts, and management of needle phobia: An international survey of a general adult population
Source: PLoS One. 2022 Nov 21;17(11):e0276814. doi: 10.1371/journal.pone.0276814 (PMC9678288; doi:10.1371/journal.pone.0276814)
Supplement: S1 Appendix — (DOCX) [file pone.0276814.s001.docx]

**Needle Phobia Questionnaire**

Assessment of Needle Phobia

*This section will identify the existence and levels of needle fear experienced by each participant and its impact on their medical care and overall well-being. This will ultimately identify those with some level of needle phobia and the underlying reasons that may contribute to this fear.*

Q1. Do you experience a fear of needles? (Y/N)

Q2. (If yes to Q1) When do you experience it? (Select all that apply):

- Before a medical procedure
- During a medical procedure
- After a medical procedure

Q3. (If yes to Q1) How long have you had needle phobia?

- < 18 years old
- 18+ years old

Q4. Has anyone in your family ever been diagnosed or reported having needle phobia?

Q5. Please rate the intensity of the needle fear you feel during, before or after medical procedures/interventions on a scale from 0 to 10, where 0 indicates ‘no fear at all,’ and 10 indicates ‘very strong, unreasonable fear or avoidance.’

Q6. Which of the following contribute to the needle fear that you experience during or before medical procedures/interventions? Please select all that apply and rank based on their role in your overall fear of needles:

- Pain
- Previous traumatic experience with needles
- General anxiety
- Having to see blood
- Fear of fainting/feeling dizzy
- Disgust regarding the procedure
- Fear of something going wrong during the procedure
- Other [please specify]

Q7. Have you ever avoided medical treatment (e.g., blood draws, injections/vaccinations) because you knew a needle would be involved? (Y/N)

Q8. (If yes to Q7) What was the main reason you avoided this procedure?

- Pain
- Previous traumatic experience with needles
- General anxiety
- Having to see your own blood
- Fear of fainting/feeling dizzy
- Disgust regarding the procedure
- Fear of something going wrong during the procedure

Q9. (If yes to Q7) Would you have undergone the treatment (e.g., blood draws, injections/vaccinations) if needles were not involved?

Q10. Which of the following procedures would you avoid in order to reduce your exposure to needles? (Select all that apply):

- - Vaccinations
- Blood draw from a vein in the arm
- Capillary blood draw (fingerstick)
- Injection for pain relief
- Injection for the treatment of a mild medical condition (low risk of morbidity)
- Injection for the treatment of a severe medical condition (significant risk of morbidity/mortality)
- Blood donation

Mitigation Strategies for Needle Phobia

*The goal of this section is to understand which mitigation strategies may be effective in reducing needle fear. This may include reducing existing barriers and identifying motivators to undergoing needle-based procedures.*

Q11. Have you ever sought help for needle-phobia?

- Yes – saw a therapist in-person
- Yes – saw a therapist remotely (e.g., video call, phone call, or text)
- Yes – took other action [Please Specify]
- No

Q12. Have you shared your fear of needles with any of the below medical personnel?

- Physician
- Nurse
- Other clinician [Please Specify]

Q13. (If yes to Q12) Please describe the provider’s response on a scale from 0 to 10, where 0 indicates ‘Unhelpful,’ and 10 indicates ‘Extremely Helpful.’

Q14. Would any of the following device-based interventions reduce your fear of needles? Please select all that apply and rank their effectiveness in addressing your fear of needles:

- Smaller/thinner needles
- Autoinjectors (i.e., invisible needles)
- Needle-free jet injectors (medicine and vaccines delivered intramuscularly or subcutaneously via a narrow, precise stream rather than through a needle)
- Insulin delivery devices (i.e., single use pens, insulin pumps)
- Non-invasive alternatives (e.g., oral medications, patches)

Q15. Would any of the following non-device-based interventions reduce your fear of needles? Please select all that apply and rank their effectiveness in addressing your fear of needles:

- Education/information on how the medical equipment works
- Distractions during the procedure (e.g., watching television, Virtual Reality (VR) headset)
- Seeing a therapist (e.g., cognitive behavioral therapy)
- Watching blood draw videos before/during the procedure
- Relaxation techniques (i.e., deep breathing)
- Consultation with the clinician regarding the importance of procedure/treatment
- Using topical numbing creams

Demographics/Background

*This section aims to identify the relevant demographic details of the respondents that may predispose them to some level of needle tolerance or phobia.*

Q16. Indicate your age:

Dropdown numerical menu (years): 18-100

Q17. Indicate your sex

- Male
- Female
- Other
- Prefer not to answer?

Q18. Where are you located?

- North America
- Europe
- Middle East
- Asia
- Oceania (Australia, New Zealand, Pacific Islands)
- South America
- Africa

Healthcare-Based Demographics

*This section aims to identify demographic characteristics of the participants, including respondents’ medical history and overall perceptions of medical care.*

Q18. Are you currently or have you previously worked as a healthcare professional?

- Yes
- No
- Prefer not to answer

Q19. Do you have any non-needle-related medical fears?

- Yes
- No
- Prefer not to answer

Q20. Do you have any conditions that frequently require injections or blood draws?

- Yes
- No
- Prefer not to answer

Q21. On a scale of 0 to 10 (10 being the highest), how would you rate your overall pain tolerance?
