# Supplementary material for: Prevalence, causes, impacts, and management of needle phobia: An international survey of a general adult population
Source: PLoS One. 2022 Nov 21;17(11):e0276814. doi: 10.1371/journal.pone.0276814 (PMC9678288; doi:10.1371/journal.pone.0276814)
Supplement: S1 Table — (DOCX) [file pone.0276814.s002.docx]

| **Variable** | **Coefficient** | **Value** |
| --- | --- | --- |
| Any non-needle-related medical fears | b | 0.76 |
| Age group | c | -0.55 |
| Needle phobia family history | d | 0.51 |
| Condition that requires frequent injection or blood draw | e | 0.36 |
| Sex | f | -0.20 |
| Household income | g | -0.18 |
| Highest level of education | h | -0.14 |
| Current or previous healthcare professional | i | 0.11 |
| Region | j | -0.07 |
