# Supplementary figures and images for: Prevalence, causes, impacts, and management of needle phobia: An international survey of a general adult population
Source: PLoS One. 2022 Nov 21;17(11):e0276814. doi: 10.1371/journal.pone.0276814 (PMC9678288; doi:10.1371/journal.pone.0276814)

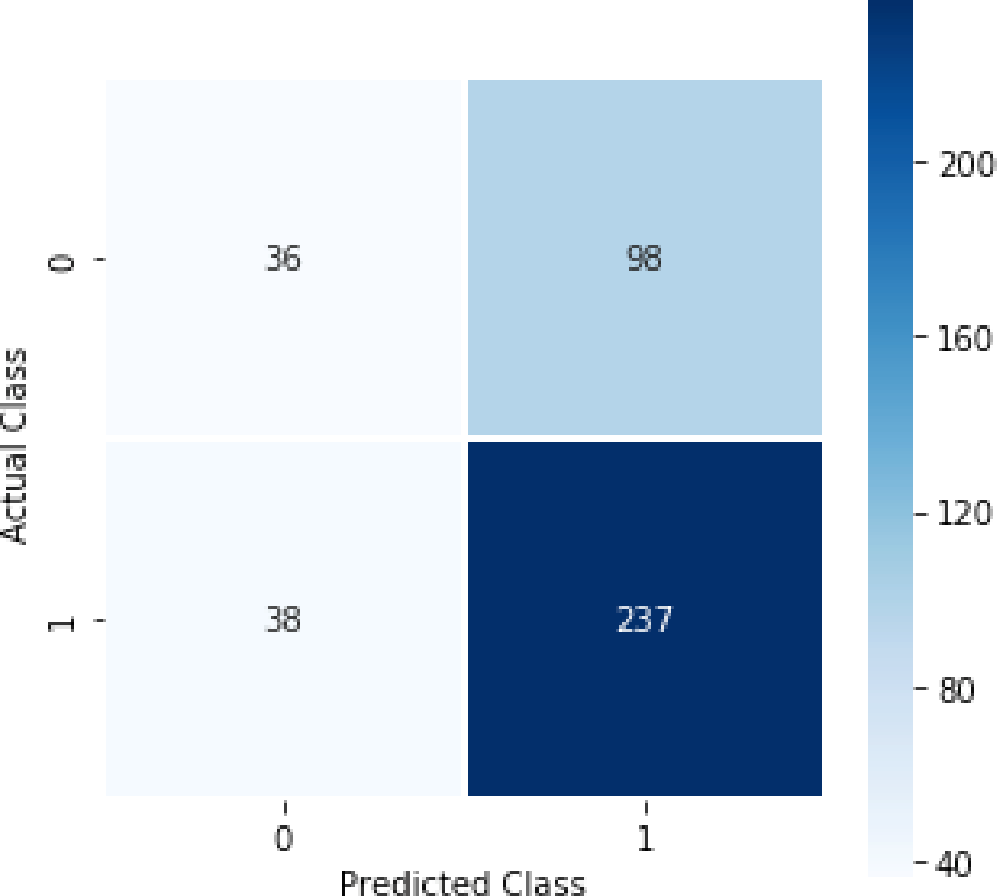

Supplement: S1 Fig — (TIF) [file pone.0276814.s003.tif]

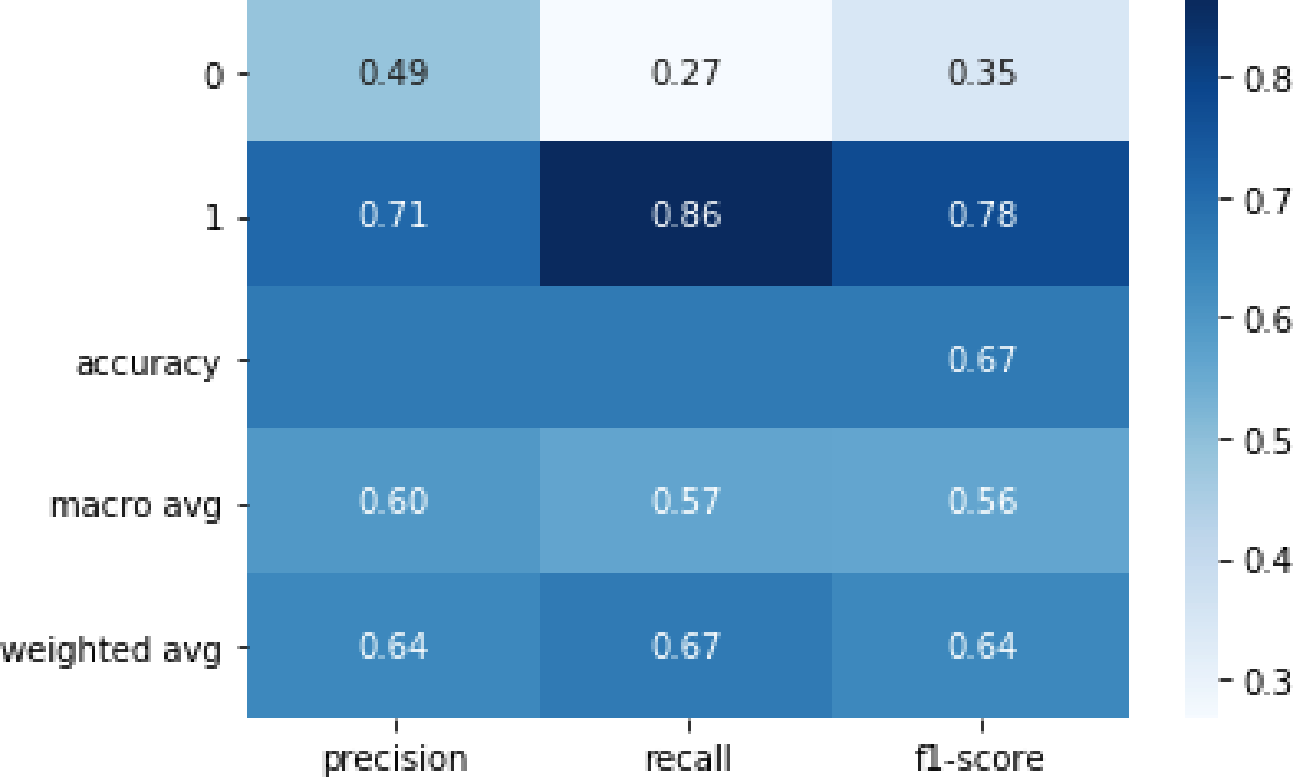

Supplement: S2 Fig — (TIF) [file pone.0276814.s004.tif]
